# The Histopathological Investigation of Red and Blue Light Emitting Diode on Treating Skin Wounds in Japanese Big-Ear White Rabbit

**DOI:** 10.1371/journal.pone.0157898

**Published:** 2016-06-27

**Authors:** Yanhong Li, Jigang Zhang, Yanfeng Xu, Yunlin Han, Binbin Jiang, Lan Huang, Hua Zhu, Yuhuan Xu, Weiling Yang, Chuan Qin

**Affiliations:** 1 Key Laboratory of Human Diseases Comparative Medicine, Ministry of Health, Institute of Medical Laboratory Animal Science, Chinese Academy of Medical Sciences, Beijing, China; 2 Key Laboratory of Human Diseases Animal Models, State Administration of Traditional Chinese Medicine, Peking Union Medicine College, Beijing, China; 3 The General Hospital of the PLA Rocket Force, Department of Dermatology, Beijing, China; Massachusetts General Hospital, UNITED STATES

## Abstract

The biological effects of different wavelengths of light emitting diode (LED) light tend to vary from each other. Research into use of photobiomodulation for treatment of skin wounds and the underlying mechanisms has been largely lacking. We explored the histopathological basis of the therapeutic effect of photobiomodulation and the relation between duration of exposure and photobiomodulation effect of different wavelengths of LED in a Japanese big-ear white rabbit skin-wound model. Skin wound model was established in 16 rabbits (three wounds per rabbit: one served as control, the other two wounds were irradiated by red and blue LED lights, respectively). Rabbits were then divided into 2 equal groups based on the duration of exposure to LED lights (15 and 30 min/exposure). The number of wounds that showed healing and the percentage of healed wound area were recorded. Histopathological examination and skin expression levels of fibroblast growth factor (FGF), epidermal growth factor (EGF), endothelial marker (CD31), proliferating cell nuclear antigen (Ki67) and macrophagocyte (CD68) infiltration, and the proliferation of skin collagen fibers was assessed. On days 16 and 17 of irradiation, the healing rates in red (15 min and 30 min) and blue (15 min and 30 min) groups were 50%, 37.5%, 25% and 37.5%, respectively, while the healing rate in the control group was 12.5%. The percentage healed area in the red light groups was significantly higher than those in other groups. Collagen fiber and skin thickness were significantly increased in both red light groups; expression of EGF, FGF, CD31 and Ki67 in the red light groups was significantly higher than those in other groups; the expression of FGF in red (30 min) group was not significantly different from that in the blue light and control groups. The effect of blue light on wound healing was poorer than that of red light. Red light appeared to hasten wound healing by promoting fibrous tissue, epidermal and endothelial cell proliferation. An increase in the exposure time to 30 min did not confer any additional benefit in both red and blue light groups. This study provides a theoretical basis for the potential therapeutic application of LED light in clinical settings.

## Introduction

Skin wounds that involve the dermis and subcutaneous tissue and are less than 20 cm in diameter usually do not require skin grafts [1]. However, since spontaneous healing of such wounds takes a relatively longer time, such wounds may benefit from interventions that promote faster healing. With the development of semiconductor technology, light emitting diode (LED) has been widely studied and applied in various industries. With the technological advances in the LED chip, sources of different wavelengths of light have been developed. This has opened new vistas for application of LED light in biomedicine.

Whelan et al. [2] performed skin wound experiments in SD rats and showed that the rats treated with LED light (wavelength: 880 nm) plus hyperbaric oxygen (HBO) had the shortest healing time. The photobiomodulation effect of other different wavelength LED lights on the skin wound was not detected. Zhao Fei et al. [3] assessed the effect of local oxygen and near-infrared LED light application to experimentally induced skin wounds in rabbits, and found that only the combined treatment achieved significant results, although the mechanism was not clear. In the study by Figurová et al. [4], LED red and blue light were found to improve healing sutured skin incisions in minipigs, but the differential effect of the two kinds of light source was not described.

The biological effects of different wavelengths of LED light are liable to vary [5]; the smaller the wavelength, the higher its frequency and poorer its bio-penetrability. Reports on the treatment of skin wound and further mechanism research by signal wavelength of LED light were few.

In this study, we investigated the effect of LED red (wavelength 630 nm) and blue (wavelength: 460 nm) light for different durations of exposure time on skin-wound healing in Japanese big-ear white rabbit. A preliminary attempt at exploring the underlying mechanisms of the effect of different wavelengths of LED light has been made with an eye for potential photobiomodulation.

## Materials and Methods

### Animals

Conventional Japanese big-ear white rabbits were purchased from Beijing Fu Long Teng Fei Marginal Farms (SYXK [Beijing] 2013–0004). Rabbits (N = 16; 2–2.5 month old; 2.5–3.0 kg; 1:1 male: female ratio) were randomly divided into two groups based on the duration of exposure of LED light: 15 min/exposure (4 male, 4 female) and 30 min/exposure (4 male, 4 female) for receiving red and blue light irradiation. Animals were housed at the ordinary animal housing facility at the Institute of Medical Laboratory Animal Science, Chinese Academy of Medical Sciences (SYXK [Beijing] 2010–0030). The animals were fed separately in home cage and provided with food and water *ad libitum*.

All animal experiments were conducted in compliance with the guidelines for animal welfare of the World Organization for Animal Health, and approved by the Institute of Animal Use and Care Committee of the Institute of Laboratory Animal Science, Peking Union Medical College (permit number: ILAS-PL-2014-005).

### Light source

LEDs (GY 225; Shanxi Guang Yu Gyled Lighting Ltd. Co.) were used to generate red and blue light (wavelengths: 630 nm and 460 nm, respectively). The red and blue LED light source both produced a power density of 50 mW/cm^2^, and each light source in 15 min and 30 min group delivered total daily energy doses of 45 J/cm^2^ and 90 J/cm^2^, respectively. The spot area of the light was 300 cm^2^.

### Rabbit skin wound model

The back hairs of animals were removed by electric hair clippers and the skin disinfected with povidone-iodine (wiping from the center to the surrounding areas twice). The animals were anesthetized with 3% sodium pentobarbital (40 mg/kg) by intraperitoneal injection. After the animal was fastened, 3 circular incisions (diameter: 1.5–2 cm) were made in the skin covering the animal’s back. The depth of wounds was extended up to the muscle fascia. Surgical wounds were created in a standardized manner, in the same region of the skin and with an equal depth in all animals. After the operation, a gentle pressure was exerted on the wound for 2 min with a clean cotton swab to prevent bleeding; the skin around the wound was cleaned with 0.9% sodium chloride solution. After 48h, the animals were administered nonsteroidal anti-inflammatory drug ibuprofen (0.1 g/day, gavage administration for 2 days) (B1220005280, Guangdong Huanan Pharmaceutical Group Co., Ltd) to alleviate pain. Intramuscular injection of penicillin (400,000 units/day, for 2 days) (H23021441, HGPF) was administered to prevent infection. The animals were subjected to a health examination once a week.

### LED light source irradiation

LED irradiation was started after the operation. Out of the three wounds inflicted per rabbit, the first wound was irradiated with red light, the second with blue light; the third group served as control and received no treatment. At the time of irradiation, the other two wounds were covered with tinfoil. The tinfoil was fixed with medical tape, and replaced immediately when folded or damaged. The wounds were clearly marked. One group of rabbits was irradiated for 15 min/exposure every day, and the other was irradiated for 30 min/exposure every day. The wounds of animals were irradiated for 21 days. The vertical distance between wound and the LED light source was 15 cm.

### Monitoring of skin lesions

After establishment of the wound model, the skin wounds were examined every day, and the wounds photographed with a camera (NikonD700, Japan).

### Calculation of the number and surface area of healing wounds

The time duration, the number and surface area of healing wounds in each group were observed. The wound area was calculated using Image J software (ImageJ v2.1.4.7, NIH, USA) (Computational Method: the wound was photographed, data entered into the computer, total area of wound was measured, non-healed area measured at each time period, and finally the healing area was calculated as total area minus non-healing area) and percent wound area healed was documented in each group.

### Histopathological examination

During the study, no instances of infection or death were observed. At 21 days, one wound in the control group showed healing; animals were euthanized with pentobarbital sodium (3%) anesthesia and inguinal artery bleeding. The wound and the adjacent tissues were fixed with 10% formalin, followed by tissues sampling, dehydration (ASP300S, Leica, Germany), paraffin embedding (EG1150H+C, Leica, Germany), and preparation of sections. Histopathological examination of hematoxylin and eosin (H&E)–stained sections was performed [6].

### Immunohistochemical and Masson staining of skin tissue

Immunohistochemical staining for endothelial cell marker (CD31, ab199012, abcam), proliferating cell nuclear antigen (PCNA) (Ki67, ab15580, Abcam), epidermal growth factor (EGF, Ls-B11905, LSBio), fibroblast growth factor (FGF, ab8880, Abcam) and macrophage marker (CD68, MD11047, MDL) in the skin wound and the adjacent tissues was performed (Protocols Paraffin sections were deparaffinised and rehydrated, antigen retrieval was done and primary antibody incubation was performed overnight at 4°C, secondary antibody was added and the reaction was seen using DAB staining. Sections were then counterstained with hematoxylin, dehydrated, and observed under the microscope) fiber hyperplasia was assessed using Masson staining [7].

### Data analysis

The images were processed by Image J software (ImageJ v2.1.4.7, NIH, USA); data were entered in Microsoft Excel and statistical analyses performed using SPSS 16.0 software (SPSS Inc., Chicago, IL, USA). Data are expressed as mean ± Standard deviation (SD). Between-group differences were assessed by single-factor one way Analysis of Variance (ANOVA); *P* < 0.05 was considered statistically significant. Stained tissue slices were analyzed using Aperio slice scanner (Aperio Technologies, Inc. Vista, CA 92081, USA); results were evaluated using Image-pro-plus software (Media cybernetics, Inc. IPP 5.0, USA).

## Results

### Observation of wound healing

**Fig 1:**
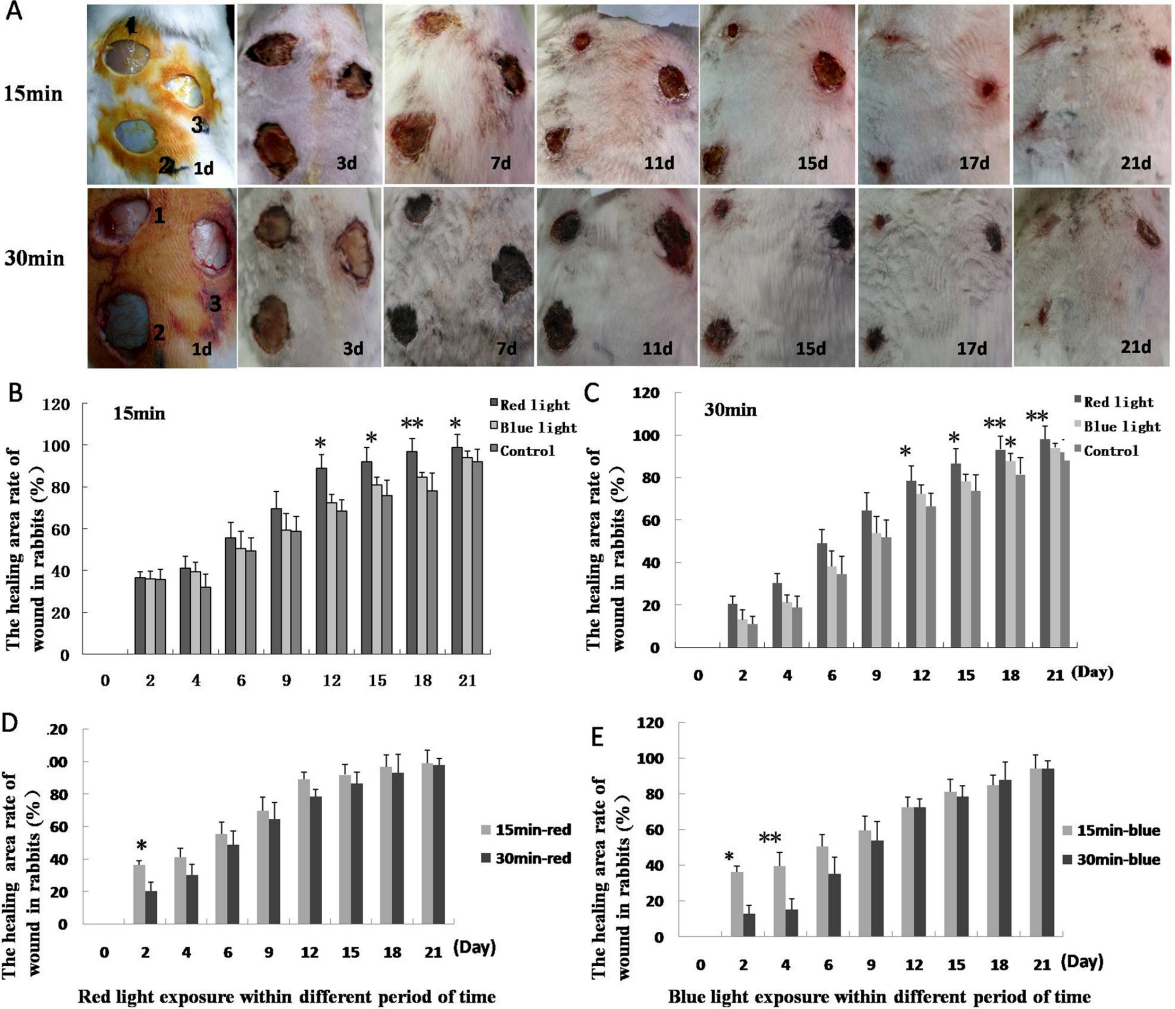
Healing of skin wounds in animals. **A**. Progress in healing of skin wounds after 15 min and 30 min duration of light exposure. 1, 2 and 3 represent red light, blue light and control groups. **B**, **C**. Healing area percentage of wounds in 15 and 30 min groups; the healing area percentage in the red light group is higher than that in control group at day 12, 15, 18 and 21 after infliction of wounds. D, E. Healing area percentage comparison between the two red light groups, and two blue light groups. The percentage in 15 min red light group was significantly higher than that in 30 min red light group on day 2. The percentage in 15 min blue light group was higher than that in 30 min blue light group on day 2 and 4 after wounds production. (*P < 0.05, **P < 0.01).

On the 3^rd^ day after the creation of wounds, scab formation was observed over the wound; tissue permeability and edema were found to have reduced, and the wounds surface area had contracted. On the 7^th^ day, the wound area in the group irradiated with red light was significantly reduced; on the 16^th^ and 17^th^ day, wounds in the 15 min red light group began healing and their scabs shed. Some wounds in the 30 min red light group began healing on the 17^th^ and 18^th^ day (Fig 1A).

### Calculation of healing wound number and area in each group animals

The time of wound healing in 15 min and 30 min blue light groups began to lessen 2–3 days later and had a prolonged healing time as compared to that in the red light group. By the time that one wound healing was observed in the control group (i.e., at day 21), four and two wounds showed healing in the red and blue light (both 15 min) groups, respectively; the corresponding number of healing wounds in the red and blue light (both 30 min) groups were both three. (Table 1). The percentage of healing area in 15 and 30 min red light groups was higher than that in other groups, and the difference on days 12, 15, 18, and 21 were statistically significant (*P* < 0.05). The percentage of healing area in the 15 min red light group was higher than that in the 30 min red light group; the between-group difference in this respect on day 2 was statistically significant (*P* < 0.05). The difference in percentage of healed wound area in the 15 min blue light group was higher than that in the 30 min blue light group on day 2 (*P* < 0.05) and 4 (*P* < 0.01) (Fig 1B–1E).

**Table 1:** The number of wounds showing healing at 21 days.

Study group	Healing wounds	Total wounds	Percentage
**15 min red light**	4	8	50%
**15 min blue light**	2	8	25%
**15 min control**	1	8	12.5%
**30 min red light**	3	8	37.5%
**30 min blue light**	3	8	37.5%
**30 min control**	1	8	12.5%

### Histopathological and immunohistochemical examination

**Fig 2:**
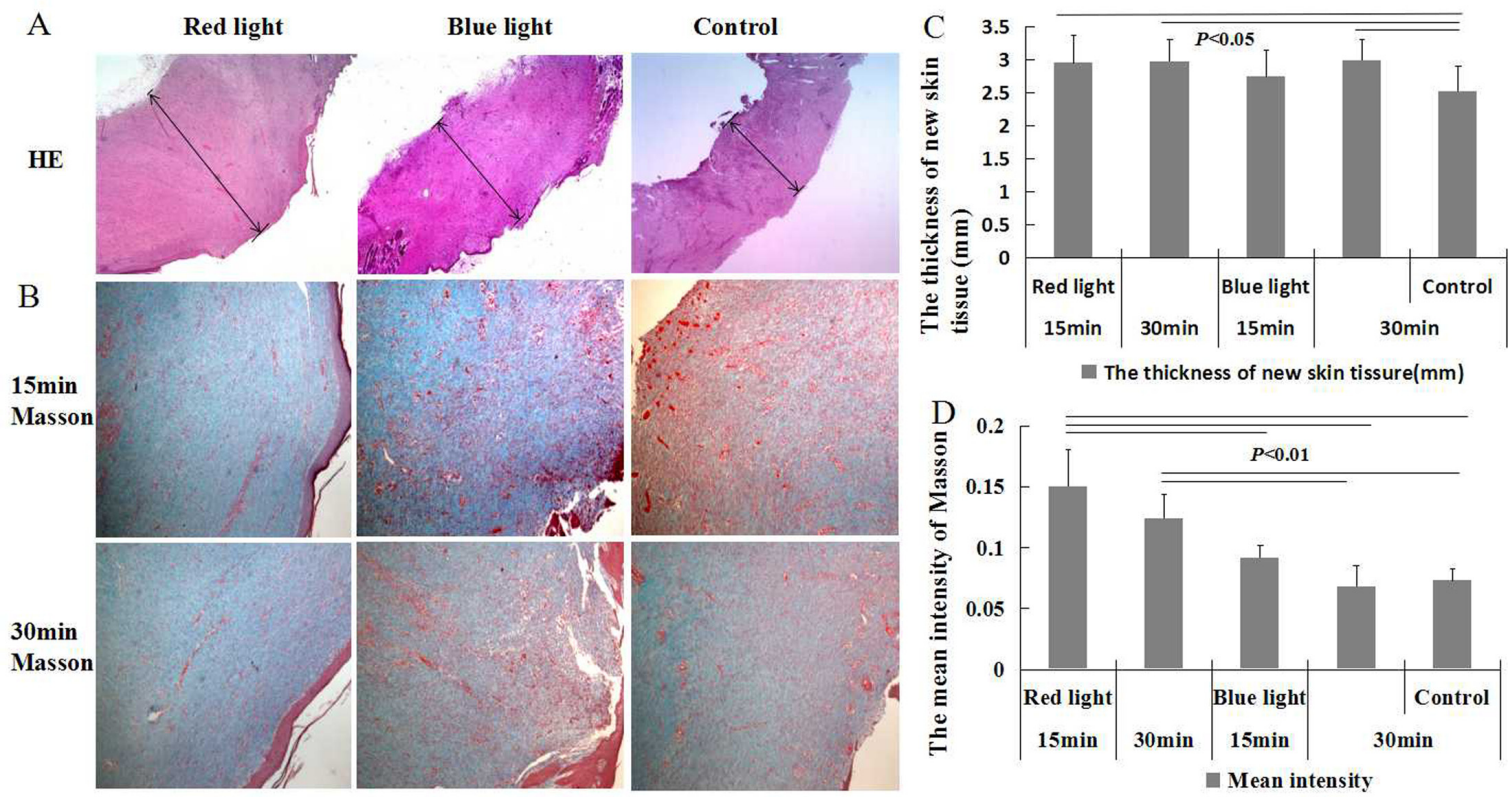
Skin HE and collagen fiber staining of animals. **A** and **B**. Skin collagen-fiber hyperplasia by study group; **C**. Thickness of new skin tissue in the red light and 30 min blue light groups was higher than that in the control group (*P* < 0.05); **D**. Mean intensity of collagen fiber in red light groups significantly increased as compared to that in the control group. (A. bar = 500 μm; B.bar = 250 μm).

The skin surface of wounds showed different degrees of scar formation. Under light microscope, different degrees of dermal fibrous tissue increase were observed (Fig 2A and 2B). Masson staining showed a significant increase in collagen fibers (*P* < 0.01) as well as skin thickness (*P* < 0.05) in the 15 min and 30 min red light groups as compared to that observed in the other groups (Fig 2). Subcutaneous hair follicles were found to have disappeared, and neovascularization, inflammatory cell infiltration, and ulcers were observed.

**Fig 3:**
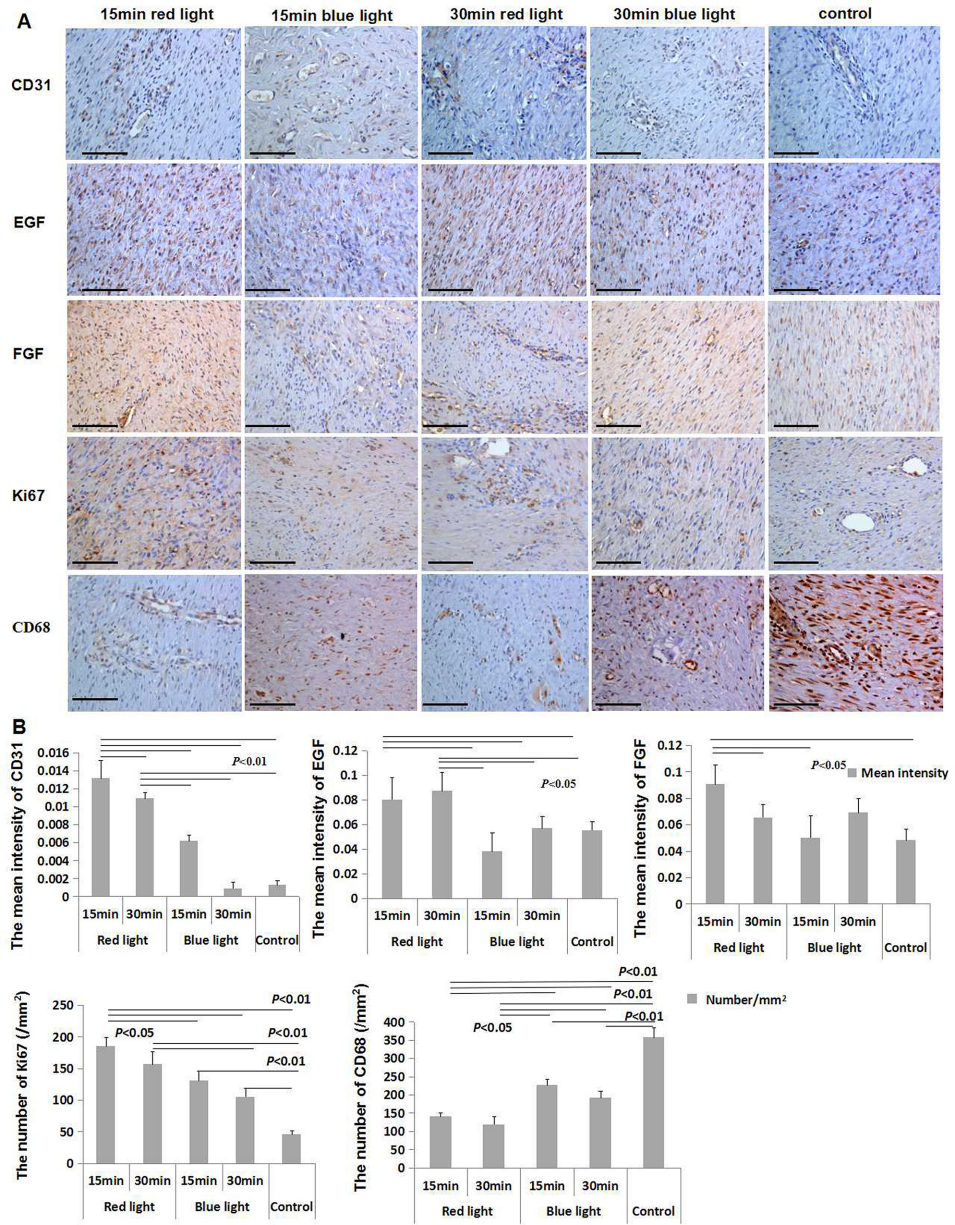
Immunohistochemical examination of animal skin lesions. **A**. Immunohistochemical staining for CD31, EGF, FEG, Ki67, and CD68 was performed in each group (Bar = 100 μm); **B**. Results showing increase in EGF level in both 15 and 30 min red light groups as compared to that in the blue-light and control group; the level of FGF in the 15 min red light group was significantly higher than that in the 15 min blue, 30 min red and the control group. CD31 level in 15 min red light group was higher than that in the other groups; that in the 30 min red light group increased compared with the blue light and control groups; the number/mm^2^ of CD68 in the red light groups was lower than that in other groups, that in blue light groups was lower than that in the control group. The number/mm^2^ of Ki67 in the 15 min red light group was higher than that in the other groups; that in the 30 min red light group was higher than that in 30 min blue light and control groups; that in blue groups were greater than that in the control group. The differences in this case were statistically significant.

Immunohistochemical examination of skin showed increased epidermal EGF levels in the 15 and 30 min red light groups as compared to that in the blue and the control groups (*P* < 0.05); the level of FGF was significantly increased in the 15 min red light group as compared to that in the 15 min blue, 30 min red and the control group (*P* < 0.05); the expression level of CD31 in the 15 min red light group was higher than all other groups (*P* < 0.01), and which in 30 min red light group was higher than that in the blue light and control groups (*P* < 0.01); the number of macrophagocyte per square millimetre in the red light groups was lower than that in blue light and control groups; that in the blue light groups was lower than that in the control group (*P* < 0.05; *P* < 0.01). The number per square millimeter of PCNA in the 15 min red light group was higher than that in the blue light and control groups (*P* < 0.05; *P* < 0.01); that in the 30 min red light group was higher than that in both 30 min blue light and the control group (*P* < 0.01); the number in blue groups was higher than that in the control group (*P* < 0.01) (Fig 3A and 3B).

## Discussion

The Japanese big-ear white rabbit was used for the skin wound model because of its relatively large skin surface area, the white color of the skin, and its similarity to the skin structure in humans [8]. These properties render it as being particularly suited to the nature of our experiment. The relatively large surface area of skin allowed for simultaneous creation of multiple wounds for irradiation with different lights. This minimized the potential for bias on account of inter-individual variability among the animals. In terms of the characteristics of the inflicted wound, we opted for removal of all skin layers (deep up to the muscle fascia) and the circular incision was about 2cm in diameter, as the healing time for this wound model is relatively long [9]. These attributes render it well-suited for evaluation of the curative effect. In our study, the wounds of control and irradiation group were in the same animal, mainly due to the individual differences in the physical condition of animals, which can affect the wound healing and confound the interpretation of the light effect. However, the light may affect the natural healing of the control wounds, so we took the method of covering the non irradiated wounds with metal foil and monitoring the irradiation process by full-time personnel to minimize the effect of light on the non irradiated wounds.

It has been suggested that near infrared low level laser can enhance skin wound healing, but such treatment require very high power density laser [10]. The advantages of LED light include its low cost, stable performance, resistance to vibration, low energy consumption, long service life, and lesser associated environmental hazards [11]. The LED light technology has advanced fairly rapidly with much improvement light intensity and peak wavelength [12]. These characteristics render LED light well-suited for diagnostic and therapeutic application.

LED red light pertains to the visible spectrum (wavelength range: 620 nm—770 nm and has the highest bio-penetrability (up to 6 mm deep from the skin surface) within commercially available LED red, yellow and blue portions of visible spectrum. The ability to directly affect the skin dermis fibroblast growth [13, 14] makes it particularly useful for skin wound treatment. LED blue light (wavelength range: 400 nm– 480 nm) has a lower bio-penetrability, with a subcutaneous penetration depth of about 1 mm, it is also used for treatment of skin disorders [5]. In this experiment, we chose red light of wavelength 630 nm, and blue light of 460 nm (power 50 MW for irradiation of the skin wounds of rabbits. LED light intensity is not uniform in all directions, and the maximum intensity is in the axial direction [15], so we chose to employ vertical irradiation. Owing to the absorption of light by the medium, the light intensity tends to decrease with increase in the distance traversed by the light. However, over a distance of < 20 cm in the air, the attenuation of light is less than 0.01% [15], so we chose to set the distance at 15 cm. In the published literature, the reported LED light treatment sessions tend to last between 10 and 30 min [15–17]. Based on this, we chose two time periods (15 and 30 min) to observe the influence of the duration of treatment on the therapeutic effect. The results showed a shorter healing time and a higher number and percent surface area of wound that showed evidence of healing in the red light group as compared to that in the blue light and the control group. No significant difference with respect to these healing parameters was observed between the 15- and 30- min sub-groups exposed to red light.

The thickness of fibrous tissue in the new skin tissue was measured. Proliferation was more apparent in the red light group as compared to that in the control group. The finding suggests a potential direct effect of red light on proliferation [11] and angiogenesis [3] of dermal fibroblasts. The mechanism is mainly through photochemical action in which the red light is absorbed by the mitochondria, and increases the activity of many intracellular enzymes, promotes cell proliferation and metabolism, and thus improves wound healing [18]. During the post-traumatic proliferation stage, vascular endothelial cells and fibroblasts, and endothelial growth factor, epidermal growth factor and fibroblast growth factor are thought to act synergistically to facilitate wound healing [19]. Active proliferation of skin histiocytes and collagen fibers was also observed in the LED red light group. Further, the collagen fibers in the scar tissue were found to be oriented parallel to the skin surface due to the effect of local tension [20]. In the proliferation stage of the skin inflammatory response, macrophage infiltration was significantly reduced in the red light group, which suggests a certain anti-inflammatory effect of LED red light, which is possibly mediated by blocking release of inflammatory factors. The underlying mechanism for this effect is not clear [21, 22, 23]. The wavelength of LED blue and red light is different, and the chromophore is also different. The absorption coefficient of haemoglobin is much higher on chromophore of blue light than other skin chromophores [24]. This particular property of haemoglobin can ensure a local temperature increase that is able to induce hemostasis through a photo-thermo-coagulation process [25]. Therefore, the blue light can perhaps influence wound healing by being absorbed by haemoglobin naturally present in these areas, causing a local temperature increase and protein denaturation within blood, resulting in a fast coagulation effect [26]. Another study showed that blue light can improve wound healing through significantly influencing biological systems, improving perfusion by release of nitric oxide from nitrosyl complexes with haemoglobin, and affecting keratin expression [27].

The skin expression levels of EGF, FGF, CD31 and Ki67 were significantly increased, and the level of CD68 expressed decreased in both 15 min and 30 min red light groups, However, on comprehensive assessment of levels of these growth factors and inflammatory factors (Fig 3B), the effect in 15 min red light group was relatively better than that in the other groups.

## Conclusions

Our study demonstrates the therapeutic effects based on histopathologic characteristics of photobiomodulation with LED red/blue light on wound healing; however, the prolongation of the duration of exposure did not appear to have a significant effect on healing. The effect of blue light irradiation was poorer than that of red light irradiation. Red light promoted proliferation of skin cells, especially those of fibroblasts, vascular endothelial cells and epidermal cells, and, thereby, accelerated wound healing. Photo-therapy is a convenient, painless and inexpensive therapeutic intervention for treatment of traumatic dermatosis. In this study, we only studied the effect of irradiation with two different wavelengths of light administered for two different durations of exposure. Further studies will focus on additional wavelengths, different power and different durations of irradiation in an effort to identify the best photobiomodulation treatment method for skin wounds.
